# Melatonin-Induced Chromium Tolerance Requires Hydrogen Sulfide Signaling in Maize

**DOI:** 10.3390/plants13131763

**Published:** 2024-06-26

**Authors:** Xiaoxiao Yang, Qifeng Shi, Xinru Wang, Tao Zhang, Ke Feng, Guo Wang, Juan Zhao, Xiangyang Yuan, Jianhong Ren

**Affiliations:** 1 College of Life Sciences, Shanxi Agricultural University, Jinzhong 030800, China; nwafu_yxx@163.com (X.Y.); sxau_zt@163.com (T.Z.); 2 State Key Laboratory of Soil Erosion and Dryland Farming on the Loess Plateau, College of Life Sciences, Northwest A&F University, Xianyang 712100, China; 3 College of Agriculture, Shanxi Agricultural University, Jinzhong 030800, China; sxau_sqf@126.com (Q.S.); sxau_wxr@126.com (X.W.); sxau_fk@126.com (K.F.); sxau_wg@126.com (G.W.)

**Keywords:** antioxidant, cell wall polysaccharide, crosstalk, H_2_S, melatonin

## Abstract

Both melatonin and hydrogen sulfide (H_2_S) mitigate chromium (Cr) toxicity in plants, but the specific interaction between melatonin and H_2_S in Cr detoxification remains unclear. In this study, the interaction between melatonin and H_2_S in Cr detoxification was elucidated by measuring cell wall polysaccharide metabolism and antioxidant enzyme activity in maize. The findings revealed that exposure to Cr stress (100 μM K_2_Cr_2_O_7_) resulted in the upregulation of *L*-/*D*-cysteine desulfhydrase (*LCD*/*DCD*) gene expression, leading to a 77.8% and 27.3% increase in endogenous H_2_S levels in maize leaves and roots, respectively. Similarly, the endogenous melatonin system is activated in response to Cr stress. We found that melatonin had a significant impact on the relative expression of *LCD*/*DCD*, leading to a 103.3% and 116.7% increase in endogenous H_2_S levels in maize leaves and roots, respectively. In contrast, NaHS had minimal effects on the relative mRNA expression of serotonin-Nacetyltransferase (*SNAT*) and endogenous melatonin levels. The production of H_2_S induced by melatonin is accompanied by an increase in Cr tolerance, as evidenced by elevated gene expression, elevated cell wall polysaccharide content, increased pectin methylesterase activity, and improved antioxidant enzyme activity. The scavenging of H_2_S decreases the melatonin-induced Cr tolerance, while the inhibitor of melatonin synthesis, p-chlorophenylalanine (*p*-CPA), has minimal impact on H_2_S-induced Cr tolerance. In conclusion, our findings suggest that H_2_S serves as a downstream signaling molecule involved in melatonin-induced Cr tolerance in maize.

## 1. Introduction

Chromium (Cr) is a prevalent heavy metal pollutant in agricultural areas, predominantly derived from industrial activities such as Cr ore smelting, leather tanning, electroplating, metal processing, and the production of paints and pigments. These activities contribute to the release of Cr-laden wastewater and exhaust gases, leading to contamination of soil and water sources [1,2]. This contamination poses a threat to food production safety, as it can enter the human body through the food chain, subsequently impacting human health [3]. The toxicity of Cr is dependent on its oxidation state, with trivalent Cr (Cr (III)) and hexavalent Cr (Cr (VI)) being the predominant forms found in soil. Cr (VI) is known to be more toxic than Cr (III) [4]. In China, a considerable portion of cultivable land, amounting to 1.26%, is presently at a notable risk of Cr pollution [5]. The severity of this contamination has led to the abandonment of 0.13% of arable land [6]. Therefore, an effective strategy to alleviate Cr toxicity in crop plants is essential.

The plant cell wall serves as the primary barrier preventing heavy metal ions from entering the cell, with cell wall polysaccharides being key components involved in the sequestration of heavy metals [7]. Cell wall polysaccharides, such as cellulose, hemicellulose, and pectin, possess functional groups such as phosphoric acid, aldehyde, and carboxyl groups that facilitate the binding of heavy metal ions [8,9]. These groups interact with metal cations to immobilize them within the cell wall, thereby limiting the penetration of metal ions into the cytoplasm. In addition, it was observed that the presence of reactive oxygen species (ROS) in plants was heightened in response to heavy metal stress, resulting in significant oxidative stress and subsequent cell damage. The research indicated that plants possess an antioxidant defense mechanism capable of regulating ROS levels [10,11]. This defense mechanism comprises the following two primary systems: enzymatic, which includes superoxide dismutase (SOD), catalase (CAT), and peroxidase (POD); and non-enzymatic, which encompasses ascorbic acid (ASA), glutathione (GSH), and flavonoids [12].

Melatonin was initially identified in the pineal gland of cattle in 1958, and subsequent research has revealed its widespread presence in both plants and animals [13]. In animals, melatonin serves various physiological and biochemical roles, such as regulating sleep patterns, antioxidative effects, involvement in stem cell differentiation, and anti-aging properties [14]. In plants, melatonin functions as a significant growth regulator and shares structural similarities with auxin, as an indole compound [15]. Melatonin plays a significant role in various physiological processes related to plant growth and development, encompassing plant morphogenesis, seed germination, and fruit ripening [16,17]. Additionally, melatonin is involved in responses to both biological and abiotic stresses, including salt stress, water stress, heavy metal toxicity, and pathogen stress [18,19,20]. Specifically, melatonin enhances plant tolerance to heavy metal stress through mechanisms such as regulation of the ASA–GSH cycle, augmentation of antioxidant enzyme activity, modulation of polyamine metabolism, and control of chelate synthesis [21,22]. Moreover, research has confirmed that melatonin plays a role in feedback mechanisms within plants, specifically in regulating various REDOX networks, such as ROS and active nitrogen (RNS), with a particular emphasis on the functions of nitric oxide (NO) and hydrogen peroxide (H_2_O_2_) [23].

In plants, H_2_S plays a significant role in the growth, development, and response mechanisms of plants to both biological and abiotic stresses [24]. This is achieved through intricate interactions with plant hormones, ROS, and various signaling molecules [25,26]. Research has demonstrated that H_2_S interacts with NO, H_2_O_2_, and mitogen-activated protein kinases (MAPKs) to increase plant resistance. Specifically, in the case of broad beans, H_2_S is implicated in the closure of stomata under salt stress conditions, acting as a downstream signal of H_2_O_2_ [27]. Furthermore, H_2_S serves as a downstream signaling molecule of both NO and H_2_O_2_ in facilitating white clover’s tolerance to drought stress regulated by spermidine [28]. In cucumbers, the protein MAPK4 functions as a downstream element of H_2_S, modulating the transcription of genes responsive to low temperatures and controlling stomatal movement during periods of low temperature stress [29]. These investigations indicate that H_2_S engages in crosstalk with additional signaling molecules, exerting a significant regulatory influence in response to abiotic stress. Nevertheless, the potential involvement of H_2_S in mediating melatonin-induced tolerance to Cr stress remains uncertain.

Maize (*Zea mays* L.), a significant staple and feed crop, exhibits diverse applications in the fields of nutrition, livestock sustenance, and various industrial sectors [30]. However, the crop’s propensity to accumulate toxic metals poses a constraint on its overall productivity within the maize production domain [31]. H_2_S has been demonstrated to play a role in melatonin-induced salt stress tolerance as a downstream signal [32], though its involvement in melatonin-induced Cr stress tolerance remains uncertain. Additionally, H_2_S and melatonin share common physiological functions in the regulation of plant tolerance to environmental stress, particularly in their involvement in the plant ROS signaling pathway [12,33]. In this study, we hypothesized that H_2_S may serve as a downstream signaling molecule involved in melatonin-induced Cr tolerance in maize. This study specifically examines the impact of H_2_S and melatonin on the regulation of cell wall Cr-binding ability and mitigation of oxidative stress.

## 2. Results

### 2.1. Response of Endogenous H_2_S and Melatonin to Cr Stress

**Figure 1:**
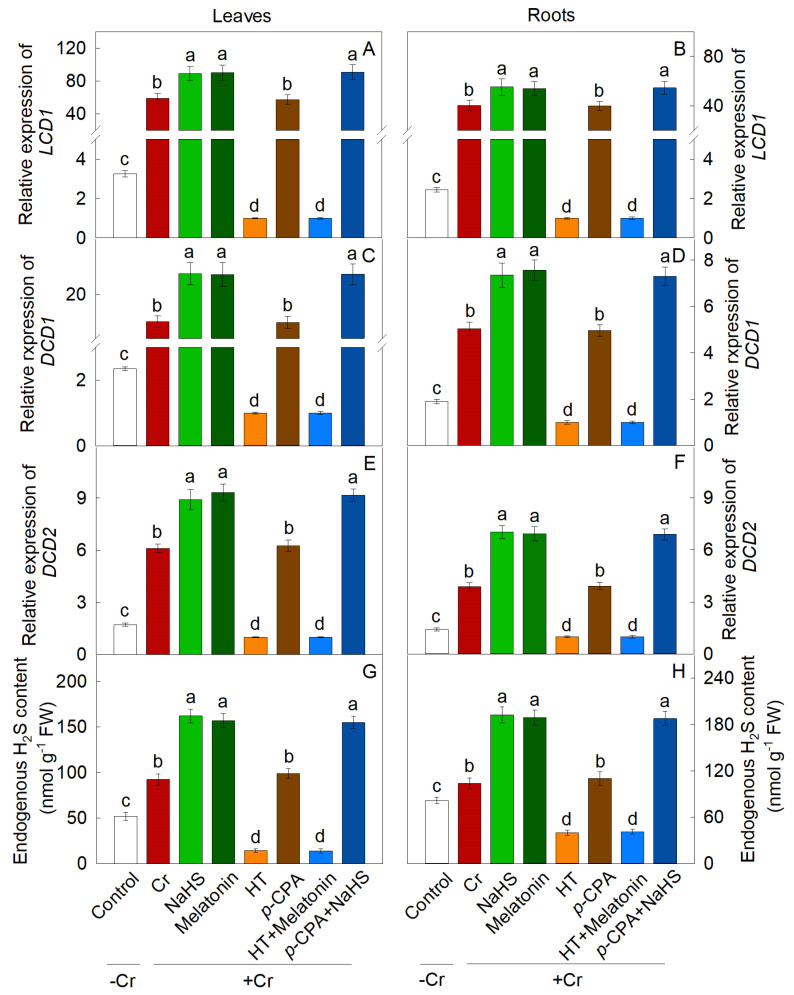
The effect of NaHS and melatonin on the expression of H_2_S biosynthesis genes (**A**–**F**) and H_2_S levels (**G**,**H**) in maize leaves and roots in response to Cr-induced stress. Different letters indicate statistically significant differences. Values are presented as mean ± SE (*n* = 3).

**Figure 2:**
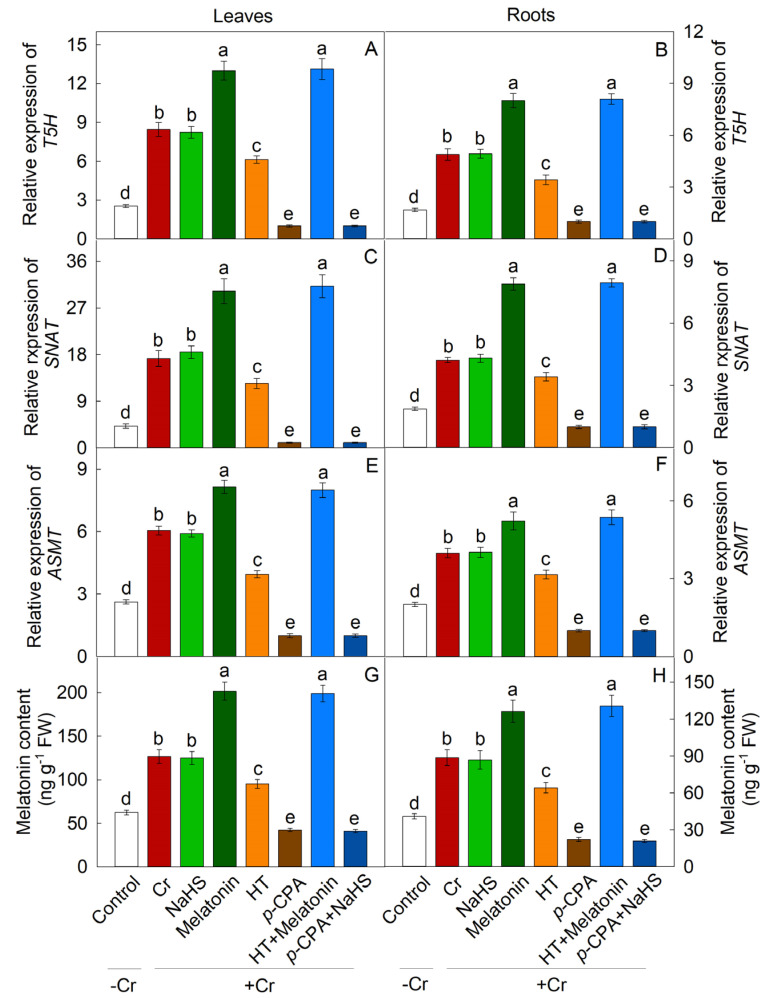
The effect of NaHS and melatonin on the expression of melatonin biosynthesis genes (**A**–**F**) and melatonin levels (**G**,**H**) in maize leaves and roots in response to Cr-induced stress. Different letters indicate statistically significant differences. Values are presented as mean ± SE (*n* = 3).

Treatment with K_2_Cr_2_O_7_ resulted in a significant upregulation of genes involved in the synthesis of H_2_S (*LCD1*, *LCD2*, *DCD1*, and *DCD2*) and melatonin (*T5H*, *SNAT*, and *ASMT*), leading to an increase in endogenous levels of both H_2_S and melatonin. Additionally, exogenous melatonin notably enhanced the transcription of genes related to L-/D-cysteine desulphurase (L-/D-CD) synthesis, and the endogenous content of H_2_S (Figure 1). Conversely, NaHS did not have a significant impact on the expression of melatonin biosynthesis genes or the endogenous levels of melatonin (Figure 2).

### 2.2. Effects of Exogenous NaHS and Melatonin on Plant Growth

**Figure 3:**
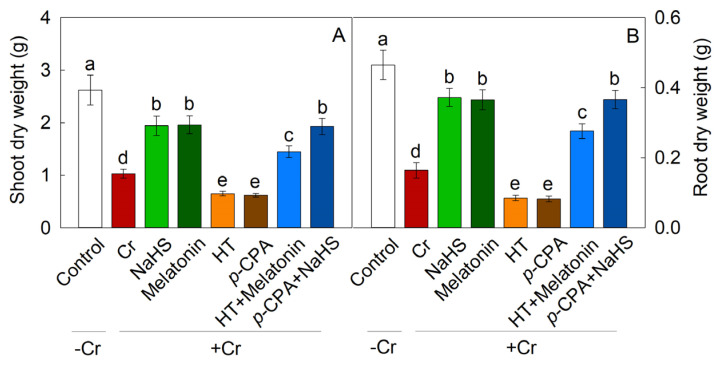
Effects of NaHS and melatonin on maize shoot dry weight (**A**) and root dry weight (**B**) under Cr stress. Different letters indicate statistically significant differences. Values are presented as mean ± SE (*n* = 3).

The inhibitory effect of Cr stress on maize growth was significantly mitigated by the addition of exogenous NaHS and melatonin, resulting in a 88.3% and 90.3% increase in the shoot dry weight (SDW), respectively, compared to the Cr stress alone. Conversely, the application of hydroxylamine (H_2_S synthesis inhibitor, HT) and *p*-chlorophenylalanine (melatonin synthesis inhibitor, *p*-CPA) resulted in a decrease in the SDW. The inhibitory effect of HT on SDW enhancement mediated by melatonin was noted, whereas the promotion of SDW by NaHS remained unaffected by *p*-CPA (Figure 3). Likewise, the alteration in root dry weight exhibited a consistent trend.

### 2.3. Effects of Exogenous NaHS and Melatonin on Cr Accumulation

**Figure 4:**
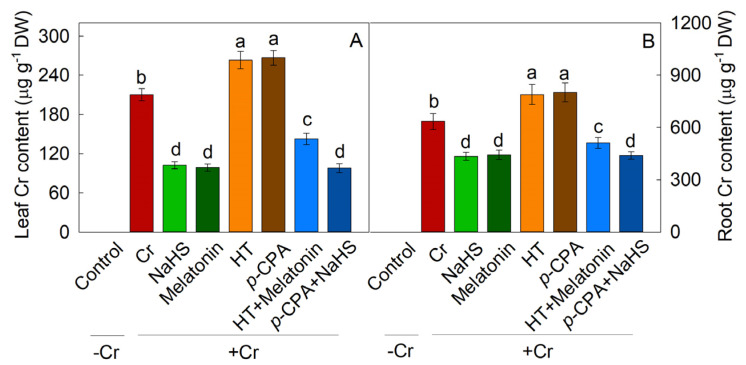
Effects of NaHS and melatonin on Cr accumulation in maize leaves (**A**) and roots (**B**). Different letters indicate statistically significant differences. Values are presented as mean ± SE (*n* = 3).

The application of NaHS or melatonin resulted in a significant reduction in Cr accumulation in maize leaves and roots. Following 7 days of Cr stress, the Cr content in maize leaves and roots decreased with the addition of NaHS and melatonin, respectively. Conversely, the Cr content increased with HT and *p*-CPA treatment. HT was found to inhibit the decrease in Cr content caused by melatonin, whereas *p*-CPA did not affect the reduction in Cr content induced by NaHS (Figure 4).

### 2.4. Effects of Exogenous NaHS and Melatonin on Cell Wall Polysaccharides’ Metabolism under Cr Stress

**Figure 5:**
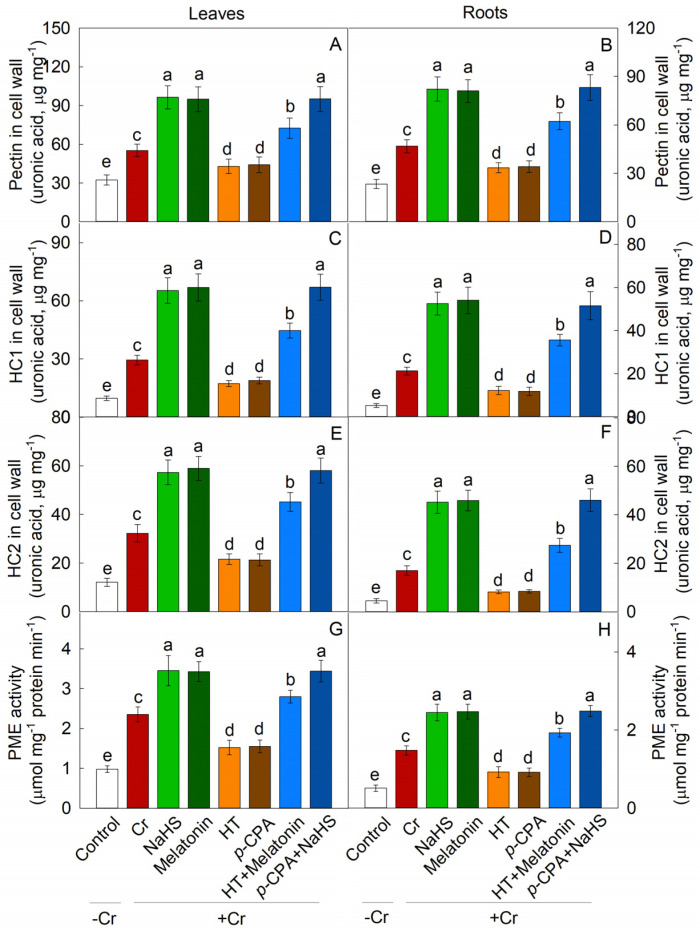
The Effects of NaHS and melatonin on the levels of pectin (**A**,**B**), hemicellulose 1 (HC1) (**C**,**D**), hemicellulose 2 (HC2) (**E**,**F**), and PME activity (**G**,**H**) in maize leaves and roots. Different letters indicate statistically significant differences. Values are presented as mean ± SE (*n* = 3).

**Figure 6:**
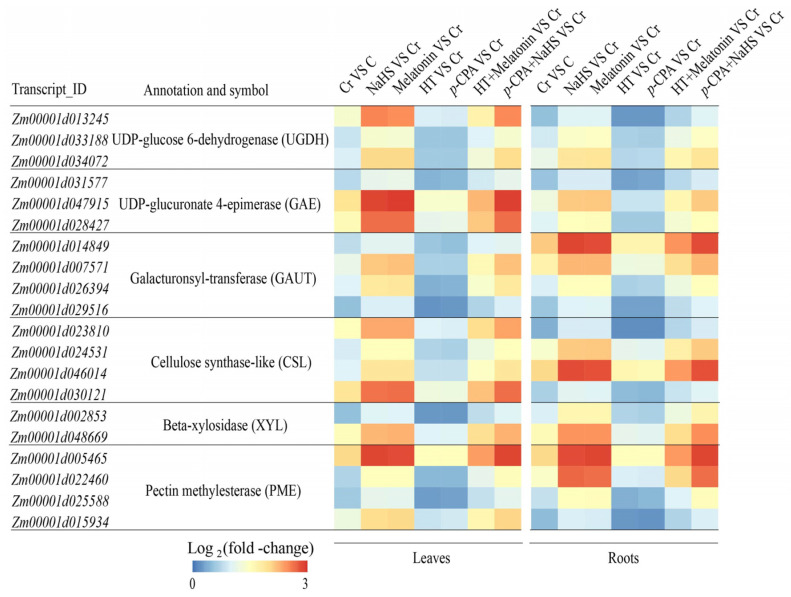
The effect of NaHS and melatonin on the expression of genes associated with cell wall polysaccharide metabolism. Scale bar denotes log_2_ (fold-change).

The addition of NaHS or melatonin resulted in a significant elevation of pectin, hemicellulose 1 (HC1), and hemicellulose 2 (HC2) levels in both maize roots and leaves (Figure 5A–F). Furthermore, the expression of pectin and HC biosynthesis genes, such as *UGDH*, *GAE*, *GAUT*, *CSL*, and *XYL*, were upregulated by NaHS or melatonin (Figure 6). Additionally, it was observed that HT hindered the melatonin-induced elevation of pectin, HC1, and HC2 levels, while the increase induced by NaHS in these components was not impacted by *p*-CPA (Figure 5A–F). The application of Cr led to a rise in pectin methylase (PME) activity. The addition of NaHS and melatonin resulted in an increase in PME activity, whereas HT and *p*-CPA caused a decrease in PME activity (Figure 5G,H). Additionally, the expression level of the *PME* gene was significantly increased by melatonin or NaHS (Figure 6). HT hindered the increase in PME activity induced by melatonin, while the increase in PME activity caused by NaHS was unaffected by *p*-CPA.

### 2.5. Effects of NaHS and Melatonin on Oxidative Damage

**Figure 7:**
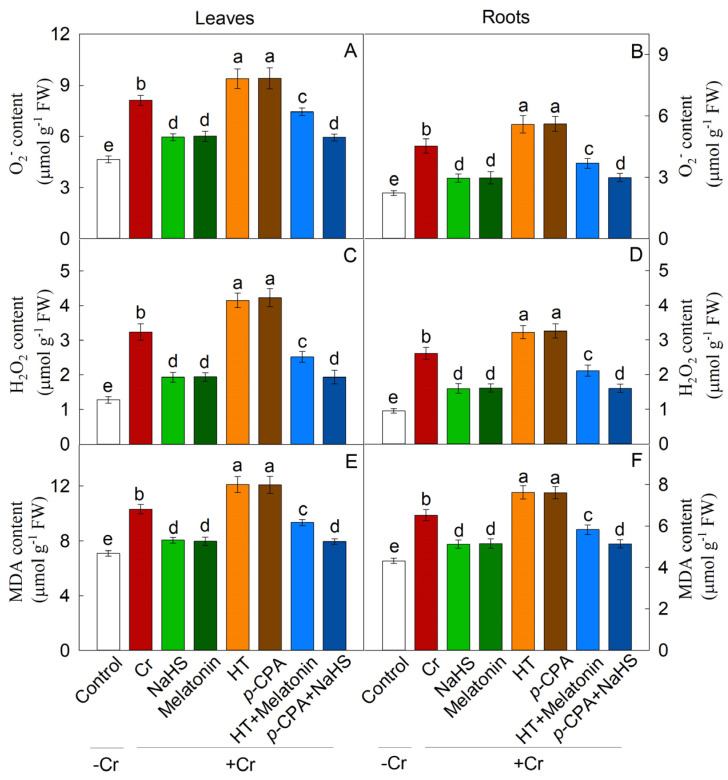
The effect of NaHS and melatonin on the levels of O_2_^•−^ (**A**,**B**), H_2_O_2_ (**C**,**D**), and malondialdehyde (MDA) (**E**,**F**) in maize leaves and roots. Different letters indicate statistically significant differences. Values are presented as mean ± SE (*n* = 3).

**Figure 8:**
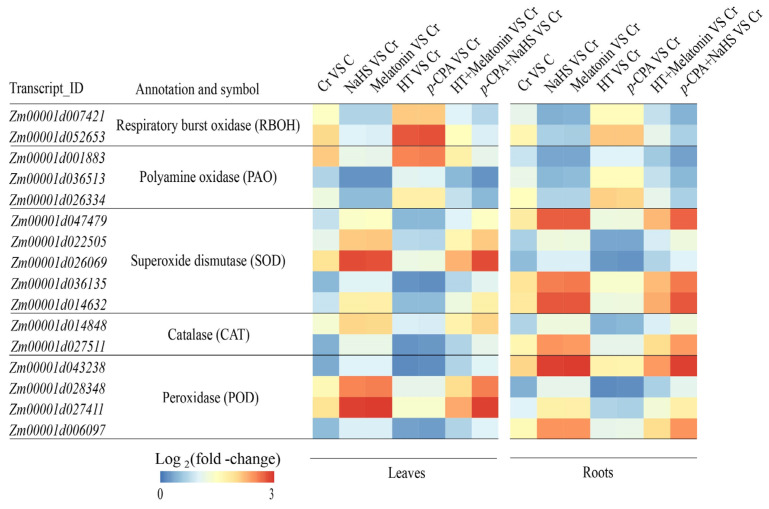
The effect of NaHS and melatonin on the expression of antioxidase synthesis genes. Scale bar denotes log_2_ (fold-change).

Cr stress resulted in elevated levels of O_2_^•−^, H_2_O_2_, and malondialdehyde (MDA) in both maize leaves and roots. Conversely, treatment with exogenous NaHS or melatonin effectively mitigated the levels of O_2_^•−^, H_2_O_2_, and MDA. In contrast, treatments with HT and *p*-CPA led to an increase in ROS content. HT was observed to hinder the reduction of O_2_^•−^, H_2_O_2_, and MDA induced by melatonin, while the reduction induced by NaHS remained unaffected by *p*-CPA (Figure 7). Likewise, the transcriptional expression of genes involved in the generation of ROS exhibited a comparable trend (Figure 8). These findings suggest that both NaHS and melatonin have the ability to mitigate ROS levels in maize seedlings, with H_2_S acting as a downstream signal of melatonin to alleviate oxidative damage caused by Cr stress.

### 2.6. Effects of NaHS and Melatonin on the Antioxidant System

**Figure 9:**
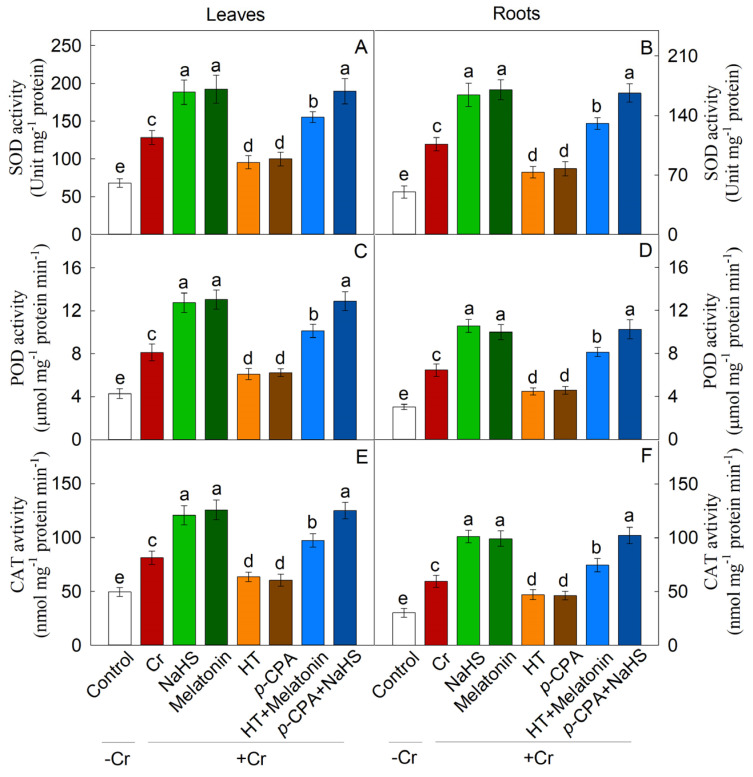
The effect of NaHS and melatonin on the enzymatic activities of antioxidants in maize leaves and roots. SOD: superoxide dismutase (**A**,**B**); POD: peroxidase (**C**,**D**); CAT: catalase (**E**,**F**). Different letters indicate statistically significant differences. Values are presented as mean ± SE (*n* = 3).

**Figure 10:**
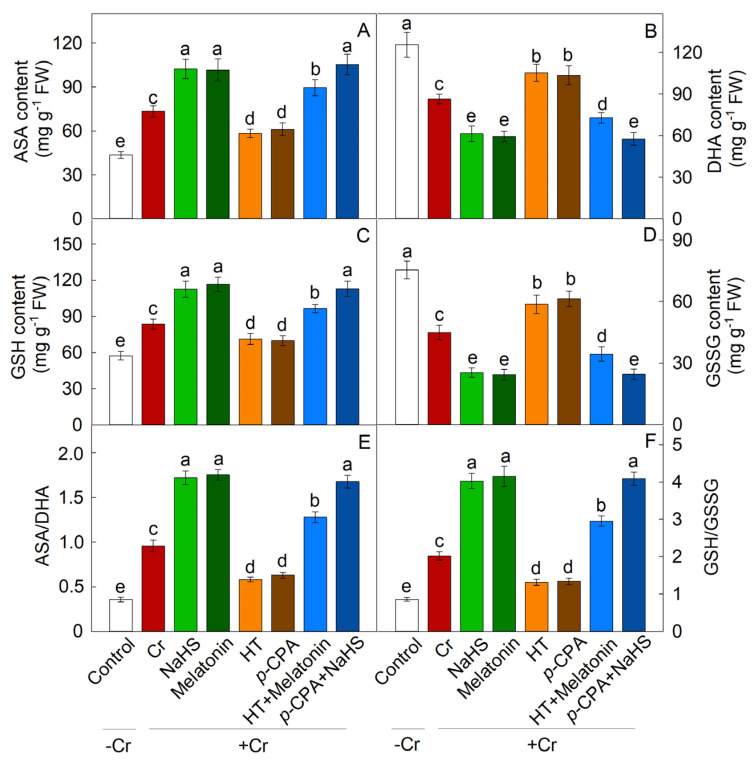
The effect of NaHS and melatonin on the levels of ascorbate (ASA) (**A**), dehydroascorbate (DHA) (**B**), glutathione (GSH) (**C**), oxidized glutathione (GSSG) (**D**), ASA/DHA ratio (**E**), and GSH/GSSG ratio (**F**) in maize leaves exposed to Cr stress. Different letters indicate statistically significant differences. Values are presented as mean ± SE (*n* = 3).

**Figure 11:**
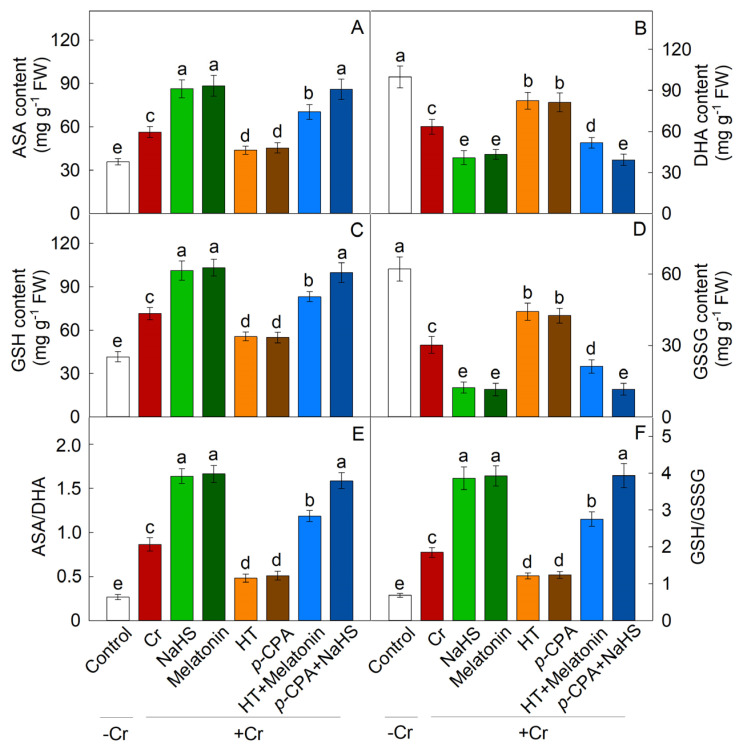
The effect of NaHS and melatonin on the levels of ascorbate (ASA) (**A**), dehydroascorbate (DHA) (**B**), glutathione (GSH) (**C**), oxidized glutathione (GSSG) (**D**), ASA/DHA ratio (**E**), and GSH/GSSG ratio (**F**) in maize roots exposed to Cr stress. Different letters indicate statistically significant differences. Values are presented as mean ± SE (*n* = 3).

Cr treatment resulted in a substantial increase in the expression of antioxidant enzymes’ synthesis genes (SOD, POD, and CAT), as well as an enhancement in enzyme activity. The addition of exogenous NaHS or melatonin notably augmented the activity of these enzymes, whereas treatments with HT and *p*-CPA resulted in a decrease in antioxidant enzyme activity. HT was found to inhibit the enhancement of antioxidase activity induced by melatonin, whereas the increase in antioxidase activity induced by NaHS remained unaffected by *p*-CPA (Figure 9). Likewise, the transcriptional expression of genes responsible for the synthesis of antioxidant enzyme exhibited a comparable trend (Figure 8). The levels of ASA and GSH were notably elevated in response to Cr stress. When compared to exposure to K_2_Cr_2_O_7_ alone, treatment with exogenous NaHS or melatonin effectively increases the levels of ASA and GSH. The presence of HT hindered the elevation of ASA and GSH levels caused by melatonin, whereas the increase in ASA and GSH levels induced by NaHS remained unaffected by *p*-CPA (Figure 10 and Figure 11).

## 3. Discussion

Previous research has indicated that H_2_S [34,35] and melatonin [36,37] have the potential to improve stress tolerance in plants. This study demonstrates that exposure to Cr stress triggers the production of endogenous H_2_S and melatonin. The growth of maize seedlings was notably impeded under Cr stress conditions, but supplementation with exogenous NaHS or melatonin alleviated this growth inhibition. The introduction of HT reversed the melatonin-induced tolerance to Cr stress, whereas *p*-CPA did not affect the H_2_S-induced tolerance to Cr stress. Through the assessment of polysaccharide content in the cell wall, levels of ROS, and antioxidant enzyme activity, it is hypothesized that H_2_S may serve as a downstream signal in mediating the tolerance of maize seedlings to Cr stress induced by melatonin.

The enzymes of LCD and DCD play crucial roles in H_2_S synthesis in plants [38]. This study observed a significant increase in endogenous H_2_S content and the expression levels of *LCD* and *DCD* in response to Cr stress. Additionally, the levels of melatonin and the expression of *SNAT* and *ASMT* were also found to increase significantly under Cr stress. Exogenous melatonin was found to markedly enhance the transcriptional activity of genes involved in the synthesis of L-/D-cysteine desulphurase (L-/D-CD) and to increase the levels of endogenous H_2_S. Conversely, NaHS did not show a significant impact on the transcriptional regulation of genes associated with melatonin synthesis or the endogenous levels of melatonin. These findings suggest that H_2_S may function as a downstream signaling molecule in mediating the tolerance of maize seedlings to Cr stress induced by melatonin.

The primary function of the root cell wall is to sequester heavy metals, thereby preventing their intrusion into the cytoplasm [39]. Among the components of the cell wall, HCs and pectin are recognized as the key elements responsible for binding heavy metals [8,40]. Higher levels of pectin and/or HC content led to increased accumulation of toxic metals in the cell walls of rice and rapeseed [9,41]. Emerging research suggests that melatonin plays a crucial role in influencing cell wall structure and composition. For instance, in tomato postharvest ripening, melatonin treatment has been shown to enhance the expression of cell wall-modifying proteins like polygalacturonase and pectinesterase. In this study, Cr treatment resulted in a significant elevation of pectin, HC1, and HC2 levels in both maize leaves and roots. Interestingly, treatment with both H_2_S and melatonin further elevated the levels of pectin, HC1, and HC2 under Cr stress conditions. The capacity of the cell wall to bind heavy metals is contingent upon the level of pectin methylation, a process regulated by PME [42,43]. Research has verified that there is a negative correlation between the accumulation of heavy metals in the cell wall and the extent of pectin methylation [44,45]. Furthermore, treatment with H_2_S and melatonin has been shown to enhance the activity of PME, facilitating pectin demethylation and increasing the number of Cr-binding sites within the cell wall. This was corroborated through experimentation involving H_2_S scavengers and inhibitors of melatonin synthesis (HT and *p*-CPA). The presence of HT had a significant inhibitory effect on the elevation of polysaccharide content in the cell wall caused by melatonin, while the increase in polysaccharide content in the cell wall induced by NaHS was unaffected by *p*-CPA.

Cr-induced stress can lead to the accumulation of ROS and can disturb the equilibrium between ROS generation and breakdown in plants. Prior research has shown that H_2_S and melatonin, acting as signaling molecules, mitigate ROS buildup and bolster antioxidant oxidase function in response to heavy metal stress [46,47]. Several researches have substantiated the function of melatonin in enhancing the activities of antioxidant enzymes, particularly under conditions of heavy metal stress [12]. The present investigation reveals that Cr stress notably triggers the buildup of H_2_O_2_ and O_2_^•−^ free radicals, resulting in oxidative damage to the leaves and roots of maize plants. The utilization of NaHS and melatonin resulted in a notable decrease in ROS accumulation in maize seedlings subjected to Cr stress. Furthermore, the application of NaHS and melatonin led to an increase in the activity of SOD, POD, and CAT enzymes under Cr stress conditions. This enhancement in enzyme activity contributed to the preservation of the cell membrane.

ASA and GSH serve as regulatory molecules in the REDOX signal transduction pathway in plant cells during abiotic stress [48,49]. The interplay between these molecules is crucial for maintaining optimal REDOX states, as plants can modulate their REDOX balance by controlling the synthesis and regeneration of ASA and GSH [50]. The ASA–GSH cycle illustrates the interconnected relationship between ASA and GSH, wherein ASA is transformed into the unstable mono dehydroascorbic acid (MDHA) free radical, leading to the production of DHA. Subsequently, DHA is converted back to ASA with the assistance of GSH as an electron donor in the reduction process [51]. Prior research has indicated that maintaining elevated ratios of GSH/GSSG and/or AsA/DHA may play a crucial role in effectively mitigating the accumulation of reactive oxygen species induced by abiotic stress [52]. Nahar et al. [53] proposed that upholding high AsA/DHA and GSH/GSSG ratios is essential to facilitate the functionality of AsA and GSH within the ASA–GSH cycle, and other physiological pathways in the presence of heavy metal stress. The results of this study demonstrate that Cr stress induces an increase in the levels of ASA and GSH, as well as the ratios of ASA to DHA and GSH to GSSG in both the roots and leaves of maize plants. Furthermore, the application of NaHS and melatonin to seedlings resulted in a further enhancement of ASA and GSH content, particularly in the ratios of AsA/DHA and GSH/GSSG, when exposed to Cr stress. These findings suggest that Cr stress disrupts the REDOX balance in plants, leading to an accumulation of DHA and GSSG. NaHS can regulate the REDOX homeostasis of ASA and GSH by increasing the ratios of AsA/DHA and GSH/GSSG, thereby contributing to the tolerance of Cr induced by melatonin. In order to further understand the significance of H_2_S and melatonin in clearing ROS under Cr stress, we conducted experiments using H_2_S scavengers and melatonin synthesis inhibitors (HT and *p*-CPA). The addition of HT significantly hindered the enhancement of melatonin’s antioxidant capabilities, while the addition of *p*-CPA did not impact the ROS clearance capacity induced by NaHS. Thus, it is hypothesized that the efficacy of melatonin may be contingent upon H_2_S signaling. While the activation of H_2_S and melatonin anti-oxidation mechanisms has been confirmed under abiotic stress conditions, the interplay between H_2_S and melatonin in the regulation of ROS metabolism under Cr stress remains poorly understood. Our investigation revealed that H_2_S plays a crucial role in mediating the tolerance of maize seedlings to Cr stress induced by melatonin, particularly in mitigating oxidative stress. Consequently, it is suggested that H_2_S may serve as a downstream signaling molecule involved in the defense mechanisms of maize seedlings against Cr stress triggered by melatonin.

## 4. Materials and Methods

### 4.1. Plant Growth and Experimental Design

The maize seeds (ZD958; Cr-sensitive) were disinfected with a 1% solution of sodium hypochlorite for 10 min, followed by three washes with distilled water. Subsequently, the seeds were placed on double filter paper and incubated at 25 °C in darkness for a period of 3 days. Subsequently, the seedlings were transferred to a plastic container containing 5 L of Hoagland nutrient solution [54], with a pH of 5.8. Hoagland nutrient solution includes KNO_3_ (6 mM), NH_4_H_2_PO_4_ (1 mM), MgSO_4_·7H_2_O (2 mM), Ca(NO_3_)_2_·4H_2_O (4 mM), EDTA-Fe (53.7 μM), CuSO_4_·5H_2_O (0.3 mM), ZnSO_4_·7H_2_O (0.8 μM), (NH_4_)_6_Mo_7_O_24_·4H_2_O (0.2 μM), H_3_BO_3_ (46.3 μM), and MnCl_2_·4H_2_O (9.1 μM). To investigate the effect of melatonin and H_2_S on Cr detoxification in maize, 14-day-old seedlings with uniform growth were subjected to foliar spraying with NaHS (50 μM), melatonin (50 μM), hydroxylamine (0.15 mM, a H_2_S synthesis inhibitor, HT), and *p*-chlorophenylalanine (100 μM, a melatonin synthesis inhibitor, *p*-CPA), 12 h before the Cr treatment. The concentration of melatonin (50 μM melatonin) used in this study was determined based on findings from our previous research [55]. Cr stress was induced by supplementing the nutrient solution with 100 μM K_2_Cr_2_O_7_ [56]. This study comprises eight treatments, including control, 100 μM K_2_Cr_2_O_7_, 100 μM K_2_Cr_2_O_7_ + 50 μM NaHS, 100 μM K_2_Cr_2_O_7_ + 50 μM melatonin, 100 μM K_2_Cr_2_O_7_ + 0.15 mM HT, 100 μM K_2_Cr_2_O_7_ + 100 μM *p*-CPA, 100 μM K_2_Cr_2_O_7_ + 0.15 mM HT + 50 μM melatonin, and 100 μM K_2_Cr_2_O_7_ + 100 μM *p*-CPA + 50 μM NaHS. The incubator conditions are as follows: temperature set at 28 °C during the daytime and 23 °C at night, photoperiod of 10 h light and 14 h dark, light intensity of 600 μmol m^−2^ s^−1^, and relative humidity ranging from 45 to 55%. Each treatment consisted of 10 pots, each containing 12 seedlings. After 7 days of Cr treatment, samples of leaves and roots were collected for subsequent analysis.

### 4.2. Measurement of Endogenous H_2_S Content

The H_2_S content was determined using the methodology described Tian et al. [57]. Root and leaf samples weighing 0.2 g were homogenized in 5 mL of phosphate buffer (50 mM, pH 6.8) containing 0.2 M ASA, 0.1 M EDTA, and 0.5 mL of 1 M HCl. The released H_2_S was captured using 1% (*w*/*v*) zinc acetate. Subsequently, 0.3 mL of dimethyl p-phenylenediamine (5 mM) dissolved in 3.5 mM H_2_SO_4_ was added, followed by 0.3 mL of ammonium ferric sulfate (50 mM). After a 15 min reaction period, the absorbance at 667 nm was measured. The standard curve was generated using different concentrations of Na_2_S. Each treatment was evaluated using three biological replicates.

### 4.3. Measurement of Endogenous Melatonin Content

The content of endogenous melatonin was quantified using the methodology described by Chen et al. [58], with the utilization of an ultra-high performance liquid chromatograph (Nexera LC-30AD, Shimadzu, Japan) and mass spectrometer (QTRAP 5500, AB SCIEX, Toronto, Canada). Fresh plant samples weighing 0.5 g were pulverized with liquid nitrogen, followed by the addition of 1 mL of pre-cooled methanol/acetonitrile/water (2:2:1, *v*/*v*/*v*) solution and subsequent vortex mixing. The resulting sample was sonicated in an ice bath for 60 min, then incubated at −20 °C for 1 h to precipitate proteins. Centrifugation was performed at 12,000× *g* rpm at 4 °C for 20 min, followed by the collection of the supernatant and subsequent vacuum drying. The dried sample was then re-suspended in a 1:1 (*v*/*v*) mixture of methanol and water (100 μL) for precipitation, and centrifuged again at 12,000× *g* rpm and 4 °C for 15 min. The resulting supernatant was collected. Following detection, the peak area of each sample was utilized as the horizontal coordinate to determine the melatonin concentration in the sample solution using a standard curve. Melatonin standard samples were procured from Sigma-Aldrich (Waltham, WA, USA), with three biological replicates measured for each treatment.

### 4.4. Measurement of Plant Dry Weight and Cr Content

In order to determine biomass, shoots and roots were collected and subsequently dried at 70 °C for 72 h before being weighed. Cr content in the roots and leaves (0.5 g) was analyzed using an inductively coupled plasma mass spectrometer (ICP-MS) (Agilent 7650A, Agilent Technologies, Santa Clara, CA, USA), following the methodology described by Sun et al. [22]. Each treatment was evaluated using three biological replicates.

### 4.5. Determination of Uronic Acid Content and Pectin Methylase Activity

Fresh tissues (roots and leaves) were extracted using 75% ethanol, and sequentially rinsed with acetone and methanol chloroform (1:1, *v*/*v*), followed by removal of the supernatant and freeze-drying of the residue. Subsequently, the pectin, HC1, and HC2 components in the resulting dried cell wall material were fractionated and isolated [59]. The quantification of glucuronic acid in each fraction was determined by utilizing a calibration standard curve established with a known concentration of galacturonic acid (GalA).

The PME activity was measured using the methodology described by Zhan et al. [39]. Fresh root and leaf samples weighing 0.2 g were collected and subsequently ground under liquid nitrogen freezing conditions. Subsequently, 5 mL of extraction solution consisting of 50 mM potassium phosphate buffer, 1 mM EDTA, and 1% PVP-30 was added to the samples. Following homogenization, the sample was centrifuged at 10,000× *g* for 20 min at 4 °C, yielding the pectin methylase extract in the supernatant. An aliquot of 8 μL of the pectin methylase extract was then mixed with 4 mL of substrate solution, and the absorbance of the resulting solution was measured at 525 nm. The concentration of H^+^ in the solution was determined to assess the activity of pectin methyl esterase. Each treatment was evaluated using three biological replicates.

### 4.6. Assays of Reactive Oxygen Species

The concentration of H_2_O_2_ was quantified using the method outlined by Loreto and Velikova [60]. Then, 0.2 g of fresh samples were put into powder under liquid nitrogen freezing conditions, and 2 mL of 0.1% (*w*/*v*) trichloroacetic acid (TCA) was added. The sample was then mixed with a buffer containing potassium phosphate (10 mM) and potassium iodide (KI) (1 M). Absorption at 415 nm was measured using a spectrophotometer (UV-2550; Shimadzu, Kyoto, Japan) to determine the H_2_O_2_ content. The O_2_^•−^ content was determined following the method described by Jahan et al. [61]. A fresh root sample weighing 0.2 g was pulverized into powder under cryogenic conditions using liquid nitrogen and subsequently combined with 2 mL of a phosphate buffer solution (50 mM) with a pH of 7.8. Following homogenization, the sample underwent centrifugation at 12,000× *g* for 20 min at 4 °C. The absorbance of the solution was measured at 530 nm. The MDA content was quantified using the method outlined by Sun et al. [22], specifically the thiobarbituric acid method. Fresh samples weighing 0.2 g were treated with 5 mL of a 0.1% TCA extraction solution. Following homogenization, the samples were centrifuged for 5 min at 4 °C at 12,000× *g*. The resulting sample solution was then combined with 4 mL of a 20% TCA solution containing 0.5% thiobarbituric acid (TBA), and heated in a water bath at 90 °C for 30 min. Absorbance readings were taken at 532 nm after the samples had cooled. Each treatment was performed in triplicate.

### 4.7. Determination of Superoxide Dismutase, Catalase, and Peroxidase Activities

For enzyme extraction, 0.2 g of fresh samples were cryogenically ground into a powder, followed by the addition of a buffer solution containing 2 mM ascorbate, 2.5 mM HEPES, 2% PVP, and 0.2 mM EDTA. The resulting mixture was homogenized and then centrifuged at 12,000× *g* for 30 min at 4 °C. The supernatant obtained from the centrifugation was utilized for the analysis of antioxidant enzyme activity. SOD activity was evaluated through its capacity to impede the photoreduction of nitrogen blue tetrazole (NBT) [62]. Prior to the quantification of enzyme activity, the incubation solution was subjected to incubation in a water bath at 30 °C. The quantity of enzymes necessary to inhibit the NBT photoreduction reaction by 50% was defined as one unit of SOD activity. The CAT activity was quantified through the decomposition of H_2_O_2_, as described by Hamurcu et al. [63]. The supernatant was combined with an incubation solution consisting of potassium phosphate buffer (25 mM, pH = 7.6), Na_2_EDTA (0.1 mM), and hydrogen peroxide (10 mM). The change in absorbance at 240 nm was monitored to determine CAT activity. POD activity was assessed by measuring the guaiacol oxidation following the addition of H_2_O_2_, following the method described by Kaya et al. [46]. Each treatment was evaluated using three biological replicates.

### 4.8. Determination of Non-Enzymatic Antioxidant Content

The determination of ASA content was conducted following the methodology described by Campos et al. [64], wherein 0.1 g of fresh sample was combined with a 5% TCA (*m*/*v*) solution. Subsequently, the mixture was homogenized and centrifuged at 10,000× *g* for 15 min at 4 °C. The resulting supernatant was then introduced into an incubation solution composed of 0.4% H_3_PO_4_ ethanol solution, 98.8% ethanol, 5% TCA (*m*/*v*), 0.5% erythrophenanthroline ethanol solution (*m*/*v*), and 0.03% FeCl_3_ ethanol solution (*m*/*v*). Absorption values at 534 nm were recorded using a spectrophotometer (UV-2550; Shimadzu, Kyoto, Japan). The content of dehydroascorbic acid (DHA) was determined by measuring the change in absorption value of dithiothreitol (DTT) at 265 nm.

The determination of GSH content was conducted following the protocol outlined by Wu et al. [65]. A fresh sample weighing 0.2 g was mixed with 2 mL of a 5% sulfosalicylic acid solution and homogenized. The resulting extracts were then centrifuged at 4 °C for 20 min at 12,000× *g*. The total GSH content was quantified by monitoring the absorption rate at 412 nm. The concentration of oxidized glutathione (GSSG) was ascertained following the elimination of GSH through derivatization with 2-vinylpyridine. GSH levels were calculated as the disparity between total GSH and GSSG. Each treatment was evaluated using three biological replicates.

### 4.9. RNA Extraction and qRT-PCR

The qRT-PCR analysis was conducted following the protocol outlined by Catala et al. [66]. Leaf and root samples weighing approximately 0.1 g and stored at −80 °C were utilized for RNA extraction using the Qiagen RNeasy^®^ Plant Mini Kit (Qiagen, Valencia, CA, USA). Subsequently, cDNA was synthesized through reverse transcription using the iScriptTM cDNA Synthesis Kit (Bio-Rad, Hercules, CA, USA). The resulting cDNA was diluted 50-fold, with 2 μL used for qRT-PCR analysis. The primer sequences can be found in Table S1. The maize *Ubi-2* gene (UniProtKB/TrEMBL; ACC: Q42415) served as an internal reference gene for the normalization of experimental data, as described by Ren et al. [12]. The 2^−ΔΔCt^ method was employed for data analysis.

### 4.10. Statistical Analysis

The data were analyzed using SPSS software (version 22) for normality testing and statistical analysis, with Sigmaplot 12.0 utilized for data visualization. One-way analysis of variance (ANOVA) was conducted within the general linear model to assess relevant indicators, and post hoc comparisons of treatment differences were made using Duncan’s tests, with a significance level set at *p* < 0.05.

## 5. Conclusions

This study indicates that H_2_S, acting as a downstream signaling molecule, plays a role in mediating the tolerance of maize to Cr stress induced by melatonin. The application of exogenous melatonin led to an increase in endogenous H_2_S levels, resulting in enhanced tolerance to Cr. This enhancement was evidenced by elevated antioxidant enzyme activity, as well as increased levels of cell wall polysaccharides and PME activity. The addition of HT decreased the Cr tolerance induced by melatonin, while the addition of *p*-CPA did not affect the H_2_S-induced tolerance to Cr. The findings of our study offer novel perspectives on the role of melatonin in mitigating Cr toxicity. Future research should incorporate molecular biology methodologies and utilize mutant materials to further elucidate the mechanisms and interactions underlying the enhancement of plant stress tolerance by H_2_S and melatonin. This study offers strategies for mitigating heavy metal toxicity and accumulation in maize, while also serving as a theoretical framework for the prevention and control of heavy metal pollution.

## Data Availability

The data are contained within the manuscript.

## Supplementary Materials

The following supporting information can be downloaded at: https://www.mdpi.com/article/10.3390/plants13131763/s1. Table S1: The primers used for qRT-PCR.
