# Supplementary material for: Melatonin-Induced Chromium Tolerance Requires Hydrogen Sulfide Signaling in Maize
Source: Plants (Basel). 2024 Jun 26;13(13):1763. doi: 10.3390/plants13131763 (PMC11244195; doi:10.3390/plants13131763)
Supplement: Supplementary file 1 [file plants-13-01763-s001.zip › plants-3052047-supplementary.pdf]

Table S1. Gene specific primers used in this study.

Transcript_ID	Annotation	Forward	Reverse
Zm00001d013245	*UDP-glucose 6-dehydrogenase*	ATCGGTGCTGGCTATGTCG	GTAGATCGGGAGGGTGTCG
Zm00001d033188	*UDP-glucose 6-dehydrogenase*	GTCTACATTTGCGAGTGCTACGGC	CGAAGCGGCTCTTCTGGTGG
Zm00001d034072	*UDP-glucose 6-dehydrogenase*	ATCGGTGCTGGCTATGTCG	GGCAGGGTGTCACTGTTCC
Zm00001d031577	*UDP-glucuronate 4-epimerase*	CGACTGGTTCGTGCGGTACTACAA	GCGGACGTGGGCATGGATTT
Zm00001d047915	*UDP-glucuronate 4-epimerase*	AGCGAGGTACAGCACCGTTCA	CATCTCCATTCCTTGGCATCTT
Zm00001d028427	*UDP-glucuronate 4-epimerase*	GACTTACAATTTGGGCAACACTTC	AACTTTCCTCACAGCCTTCACTT
Zm00001d014849	*Galacturonsyl-transferase*	TCCATCGCTTTGACACTT	GCTTACGGTCCTCATTCA
Zm00001d007571	*Galacturonsyl-transferase*	GGTGGTTATTATCATTATGCG	TGGAGTTGGTGGGTTTGT
Zm00001d026394	*Galacturonsyl-transferase*	ATTCCCATCCTCTAATCCG	GCAAGCATCAGGGTCAAA
Zm00001d029516	*Galacturonsyl-transferase*	CTTGGAGAAGGTGGTGTT	CTTGGAGCTGATGGTAGG
Zm00001d023810	*Cellulose synthase-like*	TCAGCGTCATCTCGTGCTTCTAC	CCCACTCCGTCTTGTCCTCGT
Zm00001d024531	*Cellulose synthase-like*	GCTCTTCCACCTCTACCCTTTCG	CCACCAGACGAGCACCACCA
Zm00001d046014	*Cellulose synthase-like*	GGTACGCCATTTACGGCTTCA	TGGTCAGGGTGCTCCGACAG
Zm00001d030121	*Beta-xylosidase*	GCTCTGGTTCGCCTTCTCA	TCCGACTTGCCTGTTGGAT
Zm00001d002853	*Beta-xylosidase*	TATTCTCGGTTCAGTTGCG	TGGTTGTTGCTGGGCTTA
Zm00001d048669	*Beta-xylosidase*	GTGCTCATGTCTGGCGGTCCCA	CGTCGGCAATCGCCTGTCCT
Zm00001d005465	*Pectin methylesterase*	GCAAGGACCTGCCCAAGAACG	CCGGATGCCTCGGTGATGAG
Zm00001d022460	*Pectin methylesterase*	CTTCCATCCTCGTTTCCG	TTCACCATTCGTTAGCCTTG
Zm00001d025588	*Pectin methylesterase*	GCCCTCAAGACGCTCTACTACGC	GCCTCCTTCCGTCCGATGA
Zm00001d015934	*Pectin methylesterase*	GCCGCAGGGATGGATGGAGT	GCCGCAGGGATGGATGGAGT
Zm00001d007421	*Respiratory burst oxidase*	CCCTTGGACGACAACATCAGC	GCCCTTCACGAACCACCAGTAG
Zm00001d052653	*Respiratory burst oxidase*	GGGATGAAGGAGCCGGAGTT	GAAGCTGGTGTCGGAGATTTGG
Zm00001d001883	*Polyamine oxidase*	AACCAAAGGGTCACCGAAAT	GCCGAGTGGCACTGAGATT

Zm00001d036513	*Polyamine oxidase*	GCTTGACATGCTGGAGATG	CCCGTGCTGAGGATAAAA
Zm00001d026334	*Polyamine oxidase*	GTGGAAAGAAGAGGCAATCA	GCAGGCATGTAAACAAGGA
Zm00001d047479	*Superoxide dismutase*	TGAAGGCTGTTGCTGTGC	TCTCCCTCTTGGGTGAAAA
Zm00001d022505	*Superoxide dismutase*	AATGTGACAGCGGGAGAA	TGATACCACAGGCAACACG
Zm00001d026069	*Superoxide dismutase*	AAAACATTGAGGTGGACTTG	TGGACGAGATCCCAGAAC
Zm00001d036135	*Superoxide dismutase*	TGGCATACTGTCACTCTACG	AGCCTCTGGGATATTTGG
Zm00001d014632	*Superoxide dismutase*	TCTTGGCATACTGTCACTC	CTAGGATATTTGGCTCACC
Zm00001d014848	*Catalase*	CCGAATCCAAAGACCAAT	ATGCCAACATCGTCAAAGAG
Zm00001d027511	*Catalase*	GCAAGAGCGGTTCATAACCAGA	CGGCCTGAGACCAGTTGGAG
Zm00001d043238	*Peroxidase*	GGCTGGCTGGCGGATACTTG	GGTGGATCGGAGAAGGCAGAG
Zm00001d028348	*Peroxidase*	CCGCCTCCATTTCCACGAC	AACGCCTGGGCAAGCCTCCT
Zm00001d027411	*Peroxidase*	GGCAACCAGGCGGAGAAGGA	CAGGAGACGACGCCGAAGCA
Zm00001d006097	*Peroxidase*	ACCGCCTTCGATAACGCCTAC	TGGTCCGAGGTGAACAATCCC
NM_001329666.1	*Polyubiquitin*	TGGTTGTGGCTTCGTTGGTT	GCTGCAGAAGAGTTTTGGGTACA
